# TERT Promoter Mutations as Simple and Non-Invasive Urinary Biomarkers for the Detection of Urothelial Bladder Cancer in a High-Risk Region

**DOI:** 10.3390/ijms232214319

**Published:** 2022-11-18

**Authors:** Hamid Pakmanesh, Omid Anvari, Nathalie Forey, Elisabete Weiderpass, Reza Malekpourafshar, Maryam Iranpour, Armita Shahesmaeili, Nahid Ahmadi, Azam Bazrafshan, Kazem Zendehdel, Caroline Kannengiesser, Ibrahima Ba, James McKay, Maria Zvereva, Md Ismail Hosen, Mahdi Sheikh, Florence Le Calvez-Kelm

**Affiliations:** 1 Department of Urology, School of Medicine, Shahid Bahonar Hospital, Kerman University of Medical Sciences, Kerman 7616913555, Iran; 2 International Agency for Research on Cancer (IARC), Genomic Epidemiology Branch, 69008 Lyon, France; 3 Department of Pathology, Pathology and Stem Cell Research Center, Kerman University of Medical Sciences, Kerman 7616913555, Iran; 4 HIV/STI Surveillance Research Center, and WHO Collaborating Center for HIV Surveillance, Institute for Futures Studies in Health, Kerman University of Medical Sciences, Kerman 7616913555, Iran; 5 Department of Pharmacology and Toxicology, Faculty of Pharmacy, Kerman University of Medical Sciences, Kerman 7616913555, Iran; 6 Cancer Research Center, Cancer Institute, Tehran University of Medical Sciences, Tehran 1419733133, Iran; 7 Department of Genetics, Bichat Claude Bernard Hospital, 75108 Paris, France; 8 Chair of Chemistry of Natural Compounds, Department of Chemistry, Lomonosov Moscow State University, 119991 Moscow, Russia; 9 Department of Biochemistry and Molecular Biology, Faculty of Biological Sciences, University of Dhaka, Dhaka 1000, Bangladesh

**Keywords:** bladder cancer, urinary biomarkers, non-invasive detection, telomerase, somatic mutations, TERT promoter mutations, opium

## Abstract

Bladder cancer (BC) is the 10th most common cancer in the world. While there are FDA-approved urinary assays to detect BC, none have demonstrated sufficient sensitivity and specificity to be integrated into clinical practice. Telomerase Reverse Transcriptase (*TERT*) gene mutations have been identified as the most common BC mutations that could potentially be used as non-invasive urinary biomarkers to detect BC. This study aims to evaluate the validity of these tests to detect BC in the Kerman province of Iran, where BC is the most common cancer in men. Urine samples of 31 patients with primary (*n* = 11) or recurrent (*n* = 20) bladder tumor and 50 controls were prospectively collected. Total urinary DNA was screened for the *TERT* promoter mutations (uTERTpm) by Droplet Digital PCR (ddPCR) assays. The performance characteristics of uTERTpm and the influence by disease stage and grade were compared to urine cytology results. The uTERTpm was 100% sensitive and 88% specific to detect primary BC, while it was 50% sensitive and 88% specific in detecting recurrent BC. The overall sensitivity and specificity of uTERTpm to detect bladder cancer were 67.7% and 88.0%, respectively, which were consistent across different tumor stages and grades. The most frequent uTERTpm mutations among BC cases were C228T (18/31), C250T (4/31), and C158A (1/31) with mutant allelic frequency (MAF) ranging from 0.2% to 63.3%. Urine cytology demonstrated a similar sensitivity (67.7%), but lower specificity (62.0%) than uTERTpm in detecting BC. Combined uTERTpm and urine cytology increased the sensitivity to 83.8%, but decreased the specificity to 52.0%. Our study demonstrated promising diagnostic accuracy for the uTERTpm as a non-invasive urinary biomarker to detect, in particular, primary BC in this population.

## 1. Introduction

Bladder cancer (BC) is the tenth most common neoplasm worldwide, with an estimated 573,000 incident cases and 213,000 related deaths in 2020 [1]. While the incidence and mortality rates for BC have been slightly decreasing in the developed regions, a rising trend is being observed in the developing countries (including the Middle East), where one-third of incident BC cases originate [2,3]. More than 50% of bladder cancer cases globally are attributable to tobacco use [4]. However, other specific risk factors, including chronic infection with *Schistosoma haematobium* in Africa [5], opium consumption in Iran [6,7], and specific occupational exposures [8], can also contribute to the high rates of BC in some regions.

Due to the expensive diagnostic modalities (CT-urography and cystoscopy), high recurrence rate, and the requirement for long-term follow-up, bladder cancer is considered to have the highest lifetime cost per patient among all cancers [9]. Primary non-muscle invasive bladder cancer (NMIBC) is the most common type of BC that accounts for 65–70% of all cases [10]. While NMIBCs are primarily managed conservatively [11], there is a significant risk of recurrence (up to 78%) or progression to muscle-invasive bladder cancer (MIBC) (up to 45%) [12]. Patients with NMIBC necessitate long-term follow-up with repeated cystoscopy examinations and urine cytology [11]. MIBC has a high propensity for rapid growth and distant metastasis. Its prognosis depends on staging, histology variants, and treatment. Radical cystectomy remains the recommended treatment in highest-risk non-muscle-invasive and muscle-invasive nonmetastatic BC, with a reported 5-year survival rate of about 50% for the latter [13]. The outcome for patients with MIBC could be improved with cisplatin-based neoadjuvant chemotherapy [14]. Overall, it has been shown that delayed diagnosis of BC can increase the risk of death [15]. Therefore, finding non-invasive methods for early detection and management of BC is critical to reduce its burden.

Urine is directly exposed to bladder tumor tissue, making it a unique reservoir for potential biomarkers that could be utilized to detect and monitor BCs. Intact tumor cells can desquamate into the urine as exfoliated cells (Cellular-DNA) or release small cell-free DNA fragments (cfDNA) into the urine. Most research studies to date have focused on exfoliated urinary cells [16,17]. While there are few FDA-approved urinary assays to detect BC, none have demonstrated sufficient sensitivity and specificity to be adopted by clinical guidelines to replace or complement cystoscopy for early detection or surveillance of bladder cancer [18].

In 2013, two mutations (C228T and C250T) in the promoter region of the Telomerase Reverse Transcriptase gene (*TERT*) were found and reported to be frequently occurring in multiple tumor types, including BCs [16,17,19,20]. Several recent studies from high-income countries have shown the detection of *TERT* promoter mutations in urinary DNA (Cellular-DNA and cell-free DNA), in samples which were collected closely before the diagnosis and during post-surgical follow-up, with varying sensitivity and specificity values ranging from 52–87% and 83–99%, respectively [16,20,21,22,23,24,25]. However, due to a lack of studies in low- and middle-income countries (LMICs), the clinical utility, sensitivity, and specificity of this biomarker for the detection of BC are not known in these high-risk regions. Given the high burden of BC in LMICs, and the limited access to specialized diagnostic modalities in these regions, and also the prevalence of exposure to unique bladder carcinogens in LMICS (e.g., opium in Iran), it is critical to assess the clinical utility of uTERTpm as a non-invasive biomarker for the detection of BC among these populations.

We undertook this case–control study to assess the clinical performance of uTERTpm as a non-invasive detection method for identifying BC is a high-risk region of Iran.

## 2. Results

### 2.1. Descriptive Statistics

A total of 88 patients accepted to participate in this project. Thirty-six patients had a primary diagnosis of BC and were categorized in the case group, while 54 patients who had other urological conditions were allocated to the control group. Seven patients were excluded due to losing their urine specimen (three patients in the case group) or the inability to extract DNA from their urine (two patients in the case and two in the control groups), resulting in having a final sample size of thirty-one patients in the case and fifty in the control groups for the final analysis. Of the total 31 patients in the case group, 11 (35%) were diagnosed with primary urothelial carcinoma of the bladder in transurethral resection of bladder tumor (TURBT), and 20 (65%) were diagnosed with recurrent urothelial carcinoma of the bladder in the follow-up cystoscopy.

The mean (±Standard Deviation) of the participants’ age was 61.6 (±12.2); 60 participants were male (74%), and 21 were female (26%); 34 were smokers (42%) and 31 were opium users (38%). In the case group, most patients had hematuria (91%) and NMIBC (77%) lesions, while the tumor grade was distributed equally (52% low- and 48% high-grade) across the case group. Demographics and specific exposures among the cases and controls are summarized in Table 1. There were no significant differences in participants’ age (*p* = 0.058) and sex (*p* = 0.11) between the case and control groups. However, opium use (*p* < 0.001) and smoking (*p* < 0.001) were significantly more prevalent among the case group (Table 1). Table S2 illustrates the distribution of demographics, specific exposures, and clinical features across patients with primary vs. recurrent bladder cancers. While the prevalence of opium use and smoking was higher in the primary bladder cancer group, no risk factor was statistically different among the two groups (Table S2).

### 2.2. Performance of Urinary TERT Promoter Mutations (uTERTpm) in Detecting BC

In total, 21 out of 31 BC cases (67%) and 6 out of 50 controls (12%) tested positive for *TERT* promoter mutations in urinary DNA samples. Of these *TERT* mutations, 22 were C228T (18 cases, 4 controls), 5 were C250T (4 cases, 1 control), 1 was A161C (1 control), and 1 was a newly identified C158A (1 case). The ddPCR screening assay designed to interrogate the A161C mutation identified a case with a different mutational profile as A161C indicative of a potential adjacent mutation. The C158A mutation was confirmed by Sanger sequencing (Figure S1). The C228T mutation was the most frequently detected *TERT* promoter mutation type in both case (18/31; 58.1%) and control (4/50, 8%) groups. Further, in two cases, concomitant C228T and C250T mutations were detected (Table S3).

Table 2 summarizes the performance of uTERTpm (including sensitivity, specificity, positive predictive value (PPV), negative predictive value (NPV) and accuracy) for detecting primary and recurrent BC cases. The overall sensitivity of uTERTpm for detecting primary and recurrent BC was 67.7% (95% CI: 48.6–83.3), and the overall specificity was 88.0% (95% CI: 75.6–95.4), and the overall accuracy was 81.9% (95% CI: 71.8–89.6). When we stratified the analysis by the type of diagnosed BC cases (primary vs. recurrent), uTERTpm showed significantly higher sensitivity and accuracy for detecting primary BC [sensitivity: 100.0% (95% CI: 71.5–100.0), accuracy: 91.6% (95% CI: 81.6–97.1)] than recurrent BC [sensitivity: 50.0% (95% CI: 27.2–72.8), accuracy: 76.6% (95% CI: 64.9–85.9)] (Table 2).

The sensitivity of uTERTpm ddPCR assays for detecting BC was consistent across all tumor stages (66.7% for NMIBC vs. 71.4% for MIBC) and grades (62.5% for low-grade vs. 73.3% for high-grade tumors) (Table 3). The uTERTpm assay tended to have a better performance in detecting BC in patients with gross hematuria and those who use opium. (Table 3).

**Figure 1:**
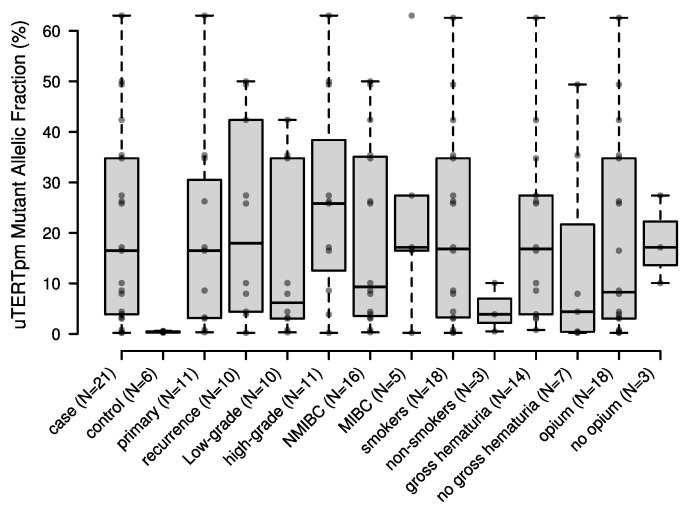
Distribution of uTERTpm mutant allelic fractions in BC cases and controls and across tumor types, grades, stages, smoking, and opium status, and gross hematuria. Box plot showing the median (solid lines), data points (circles), the 1st and the 3rd quartiles of mutant allelic fractions. The upper and lower Tukey whiskers are shown (dashed lines), i.e., whiskers extend to data points that are less than 1.5 × Interquartile range away from 1st/3rd quartile. NMIBC: non-muscle-invasive bladder cancer; MIBC: muscle-invasive bladder cancer.

Interestingly, the mutant allelic fraction (MAF) was <1% in all six false-positive samples (from the control group), whereas among the BC case group with positive uTERTpm, only 14% (3 cases out of 21) had MAF < 1%. If the detection threshold of the assay is set to MAF > 1%, the sensitivity of uTERTpm in detecting BC would slightly decrease to 64.2% (95% CI: 44.0–81.3), but the specificity would increase to 100.0% (95% CI: 92.9–100.0). Among the BC cases with positive uTERTpm, we did not observe significant differences in the MAF between primary vs. recurrent BC (*p* = 0.88), those with vs. those without gross hematuria (*p* = 0.23), and opium users vs. non-users (*p* = 0.56) (Figure 1). We observed slightly higher MAF among MIBC, high-grade BC, and smoker patients than NMIBC, low-grade BC, and non-smoker patients; however, none of these differences were statistically significant (*p* = 0.50, *p* = 0.43, *p* = 0.22, respectively).

### 2.3. Performance of Urine Cytology Alone and in Combination of uTERTpm in Detecting BC

Overall, 21 out of 31 BC cases (67.7%), and 19 out of 50 controls (38%) had positive urine cytology on recruitment. The overall sensitivity of urine cytology for detecting primary and recurrent BC was 67.7% (95% CI: 48.6–83.3), and the overall specificity was 62.0% (47.1–75.3). Urine cytology had higher sensitivity in detecting MIBC [sensitivity 100% (95% CI: 59.0–100.0)] and high-grade tumors [sensitivity 80% (95% CI: 51.9–95.7)] (Table 3).

Comparing the performance of urine cytology and uTERTpm in detecting BC in this population, indicates that urine cytology could be more sensitive than uTERTpm in detecting MIBC, while uTERTpm could be more sensitive than urine cytology in detecting BC in non-smokers (Table 3). However, the sample size was too small to allow a reliable assessment of these differences.

Combined uTERTpm and urine cytology enabled the detection of 26/31 cases which resulted in having higher sensitivity [83.8% (95% CI: 66.2–94.5)] but lower specificity [52% (95% CI: 37.4–66.3] than using only uTERTpm or urine cytology alone (Table 3). 

## 3. Discussion

In this case–control study, we evaluated for the first time the clinical performance of uTERTpm as a urinary biomarker for the detection of primary and recurrent BC in the Kerman Province of Iran, where the highest incidence of the disease in the country has been observed [26]. Analysis of the whole urine samples from 31 cases and 50 age- and sex-matched hospital-based controls using the ddPCR assay, showed that uTERTpm was 100% sensitive and 88% specific to detect primary bladder cancer, while it was 50% sensitive and 88% specific in detecting recurrent BC in this population. The overall sensitivity and specificity of uTERTpm in detecting any BC were 67.7% and 88%, respectively. The results were comparable across low-grade and high-grade tumors, as well as MIBC and NMIBC. 

We screened total urine samples that contain DNA from two sources (cfDNA and Cellular-DNA) for the presence of uTERTpm (C228T, C250T, C228A, CC242-243TT, and A161C mutations) using the established sensitive ddPCR assays. The limit of detection for the ddPCR assays is reported to be as low as 0.2% MAF in other studies [24,25], which was validated in our study. The distribution of tumor stages (77% NMIBC and 23% MIBC) and grades (equal distribution of low-grade and high-grade tumors) among BC patients from the Kerman province of Iran is similar to that of BC patients in western countries [27]. However, the main BC risk factors differ in this population, with a high prevalence of opium consumption (77%) and tobacco smoking (83%) among BC cases in Iran. Despite the differences in BC risk factors, the two most predominant uTERTpm mutation detected in the urine of BC cases in Iran were the C228T and C250T, which is similar to reports from western countries [16,28]. It has been previously shown that, in urothelial cancer as well as in other cancer types, these two C to T transitions mutations occurring at 124 bp and 146 bp upstream of the translation start codon generate new GGA(A/T) motifs that will allow E-twenty-six (ETS) transcription factors to bind, causing upregulation of TERT transcription and increased telomerase activity [29].

Although in this study we could not detect other rare uTERTpm in BC cases, (CC242-243TT, C228A, and A161C mutations) that were previously described in European studies [30,31], we have previously detected two of these rare mutations in pre-diagnostic urine samples of Iranian individuals who later developed bladder cancer [32]. The rarely observed C158A mutation that we identified in one recurrent BC case was also described as a rare mutation in bladder tumors by Huang et al. who screened uTERTpm in 799 tumor tissues of 13 different tumor types from Chinese cancer patients [30]. Therefore, differences in the geographical location and risk factors of BC do not seem to affect the patterns of uTERTpm mutation patterns in BC patients from different populations.

The overall uTERTpm sensitivity was 67.7%, which was consistent across tumor grades and stages. However, the sensitivity of this biomarker reached 100% for the detection of primary BC cases and was 50% for detecting recurrent BC tumors. The overall specificity of uTERTpm against patients with urological pathologies other than BC was 88%. Other studies have also reported an equal prevalence of uTERTpm among bladder stages and grades [16,31]. Although similar detection rates for recurrent BC cases (42% to 74%) were reported in studies that used alternative assays to identify the presence of uTERTpm in DNA of urine cell sediments (cellDNA), the sensitivity of uTERTpm for detecting primary BC was higher in our study than previous studies (100% vs. 55%–86%) [16,17,24,25,33,34,35]. However, the difference in detecting primary or recurrent BC has not been observed in the French DIAGURO case–control study, which reported equal diagnostic accuracy in both types of BC [31]. The DIAGURO study also reported a higher overall sensitivity for uTERTpm (C228T and C250T) in detecting primary and recurrent BC in urine cfDNA and cellDNA [31]. The same authors reported a decreased uTERTpm sensitivity (68%) in a validation set of primary BC cases from Portuguese retrospective urinary cellular-DNA samples [31].

While the number of primary BC cases in our study was very limited and validation in larger studies in Iran is needed, the observed high sensitivity (100%) and specificity (88%) in this population, which falls within the range of previously reported specificities (83% to 100%) in healthy or hospital-based controls in European studies [21,22,24,31,32,33], holds great promise of using a simple and non-invasive uTERTpm test for the early detection of primary BC in LMICs. This is further supported by a recent study conducted by Hosen et al. who investigated for the first time the potential of uTERTpm as a BC early detection biomarker in asymptomatic individuals using a nested case–control study within a longitudinal population-based prospective cohort of 50,000 individuals in Iran [32]. In their study, Hosen et al. assessed uTERTpm in baseline urine samples from participants who later developed primary BC in the follow-up and matched healthy controls and showed that this biomarker could be detected up to 10 years before clinical diagnosis of primary BC, with a sensitivity of 46.67% and a specificity of 100%, which shows the early occurrence of these somatic genetic changes in the development of bladder cancer and thus its suitability for early detection [32]. 

The single gene ddPCR-TERT assays used in our study demonstrated almost comparable diagnostic accuracy compared to the UroSEEK multiple markers assay (including TERT C228T and C250T mutations and other regions of interest of 10 other somatically mutated genes) for the detection of primary urothelial cancers (sensitivity of 100% versus 83%; specificity of 88% versus 95%) [25]. The simplicity of the ddPCR-TERT assays over other complex urine tests such as the UroSEEK or the test developed by Dahmcke et al. [22], that combines DNA mutation testing (TERT and FGFR3) and methylation biomarkers (SALL3, ONECUT2, CCNA1, BCL2, EOMES, and VIM), would considerably ease the clinical implementation of uTERTpm testing into routine clinical practice for the early detection of BC, especially in LMICs where access to cystoscopy facilities may often represent a barrier to early BC diagnosis. In addition, the clinical utility of uTERTpm testing for the non-invasive early detection of BC is further supported by the observed substantial increase in incidence rate of BC in some low-resource regions, such as east Asia, north Africa, and the Middle East [36].

In this study, we also demonstrated for the first time that the uTERTpm testing is relevant for the detection of BC in the context of opium consumption which is a bladder carcinogen that is widely used in Iran [7]. The sensitivity of uTERTpm in opium users tended to be higher (75.0%) than in non-users (42.8%). These findings further confirm that *TERT* promoter mutations can be detected in all types of BCs, regardless of their stage, grade, or risk factors such as age, sex, or tobacco consumption, as described in other studies [16]. Although evidence from our study combined with other international studies indicates that uTERTpm could be a universal biomarker for detecting various subtypes of BC in different populations, it is not clear whether the differences in the reported detection rates for uTERTpm in different studies originate from pre-analytical procedures reported to urine sample collection, storage, and preparations, or from urinary DNA sources (cfDNA, cellular-DNA, or total urinary DNA), or differences in the prevalence of *TERT* promoter mutations in BC across populations [20]. This issue needs to be further investigated in large international multicentric studies.

In this study, rather than screening the two types of urinary DNA (cfDNA and cellular-DNA) independently, we screened total urine DNA that includes DNA from both sources. Previous studies have shown high concordant TERTpm status in urinary cfDNA and cellular-DNA [33,34], while Avogbe et al. reported the highest sensitivity for the combined source of DNA and highlighted the added value of whole urine with both DNA sources in a few rare cases where the mutation(s) could be detected in only one of the two sources of DNA [31]. Our strategy to screen for uTERTpm in total urinary DNA (combined cfDNA and cellDNA) presents the advantage to perform only one test as opposed to two independent analyses for the two types of DNA. However, as cellular-DNA yields from urinary exfoliated cells are often higher than cfDNA yield isolated from the same total urine sample [31], in rare situations, a sample with low-level uTERTpm in cfDNA may only result in a false-negative if the low MAF is sufficiently decreased by the high amount of wild-type cellular-DNA and under the detection limit of the method. 

In our study, false-positive results for uTERTpm have been observed in 6 out of the 50 controls (4 with C228T, 1 with C250T, and 1 with A161C), all with MAF <1%. The case–control study nested within the prospective Golestan cohort study has demonstrated the detectability of uTERTpm up to 10 years prior to diagnosis of BC [32], and therefore, the early occurrence of the TERTpm in the bladder carcinogenesis. In our study, the hospital-based control group underwent diagnostic cystoscopy to rule out suspicious bladder lesions, but we could not exclude that small pre-cancer lesions reflected by MAF <1% may have been missed during the cystoscopy and that controls with low uTERTpm may be diagnosed with BC later on. However, it should be noted that 3 cases out of 20 also had uTERTpm MAF <1% and were diagnosed by cystoscopy, either indicating that clones with TERTpm were not the predominant BC clones in these cases or that the tumors only released very few TERT mutated clones or cfDNA. Another potential explanation could lie in the rare occurrence of TERTpm in leucocytes, a phenomenon called clonal hematopoiesis which has been already described by Avogbe et al. in three BC cases and one control [31], and which could reflect the detection of uTERTpm in leucocyte-rich urine samples. Interestingly, after observing almost 50% of pre-diagnostic urine samples that contained uTERTpm in the Golestan prospective cohort study, the authors screened the baseline leucocyte DNA samples of the entire case–control series to control for the occurrence of TERTpm post-zygotic germline mosaicism or clonal hematopoiesis and did not observe any [32]. This may not be surprising given that clonal hematopoiesis has been associated with increased age and that samples were screened at enrollment in the population-based study. Unfortunately, we could not screen the leucocyte DNA in the Kerman series and therefore could not control for the rare occurrence of TERTpm clonal hematopoiesis. Large studies are required to better evaluate the frequency of the TERTpm clonal hematopoiesis and its potential role in bladder cancer or its implication in the interpretation of a TERT-positive test [31]. Regardless of the origin of the uTERTpm positivity in the controls, setting the minimum MAF at 1% to consider a positive test enabled a 100% specificity but a decreased sensitivity (58.1%). Large case–control or screening studies will provide information on whether a minimum mutational load (uTERT MAF) in urine samples rather than mutational status preferably correlates with a positive imaging-based diagnosis.

The overall sensitivity for urine cytology was comparable to the uTERTpm sensitivity in our study (67.7%), but the specificity was poorer (62.0% versus 88%) and was even lower than the reported specificities for urine cytology in other studies (73–100%) [37]. Surprisingly, the observed sensitivity of urine cytology for the detection of low-grade BC (56.2%) was higher than that of previous studies, where it repeatedly showed poor sensitivities [31,35,38], suggesting that, despite recent effort to better classify the urine cytology results as per The Paris System guidelines [39], subjectivity and lack of uniformity in reporting the results of cytology examinations still exist and contribute to the limited utility of urine cytology for the diagnosis of BC. Combined uTERTpm assays and urine cytology improved sensitivity to 83.8% but significantly hampered the specificity, which dropped to 52.0%, arguing in this context against the wide use of the two combined urine markers.

While our study represents the first study to estimate the utility of the uTERTpm biomarker for the detection of BC in opium users, there are some limitations which mainly include the small number of the recruited cases and controls, reflected in the lack of statistical power to compare the performance of uTERTpm in different BC subtypes and strata. Despite promising initial results, the diagnostic accuracy of the uTERTpm for the diagnosis of BC in the Kerman province should be further assessed in large validation studies. 

## 4. Materials and Methods

### 4.1. Study Population

This case–control study was conducted at the urology department of the Shahid Bahonar teaching Hospital, Kerman, Iran, from December 2018 to January 2021. The case group included patients aged 18 years or more with primary or recurrent histologically confirmed bladder cancer, who were referred for cystoscopy and tumor resection. The control group consisted of sex- and age-matched individuals with no history of bladder cancer who underwent diagnostic cystoscopy before surgical resection of the prostate (prostatectomy) or Percutaneous Nephrolithotomy (PCNL) for kidney stone. Exclusion criteria were inability to provide a urine sample, and inability to extract DNA from the provided urine sample. Written informed consent was obtained from all participants upon recruitment into the study. The research protocol was approved by the Ethics Committees of the Kerman University of Medical Sciences (Reference number: IR.KMU.AH.REC.1398.102 [40], and the International Agency for Research on Cancer (IARC-WHO, reference number: 15-23-A2).

### 4.2. Study Design

Upon recruitment, the participants were interviewed by trained staff to fill a structured questionnaire to collect information on demographics, various exposures (including smoking and opium use), and clinical characteristics of the current diseases. We defined smokers and opium users as participants who smoke at least one cigarette per week or consumed opium for more than six months [41]. Then, the participants were asked to provide a midstream urine sample. Because all patients were referred due to pathological urologic conditions, they underwent diagnostic white light cystoscopy. Patients with mucosal lesions that suggest bladder cancer in cystoscopy underwent transurethral resection of bladder lesion (TURB), where tissue samples were collected. The control group also underwent diagnostic cystoscopy to rule out suspicious bladder lesions. An expert pathologist examined the tissue samples to determine the type of lesion (benign or malignant), and the histological subtype, stage, and grade of the bladder tumors. Patients with histologically confirmed bladder cancer were categorized in the case group, while those who did not have any suspicious lesions in the cystoscopy or no confirmed bladder cancer in the pathologic examination were categorized in the control group. 

### 4.3. Preparation and Storage of Urine Samples

Within two hours of urine collection, urine samples were separated into 15 mL Falcon^TM^ conical tubes. A total of 14 mL whole urine was combined with 1 mL conditioning buffer (980 µL) in each tube. The samples were then stored at a temperature of −80 °C. The combined conditioning buffer stabilized the urine components and molecules (including cells and DNA) for up to a month at room temperature. It also allowed the isolation of total urine DNA (cellular-DNA and cfDNA) within the same fraction. Within the first 12h of urine collection, one urine sample was sent for urine cytology examination by a pathologist blinded to the samples’ case–control status. The frozen urine samples were sent in dry ice to the International Agency for Research on Cancer (IARC) in France for uTERTpm analysis. 

### 4.4. Detection of TERT Promoter Mutations Using ddPCR Assay

DNA from total urine samples preserved with conditioning buffer were isolated using the Zymo Quick-DNA™ Urine Kit and quantified by QubitTM dsDNA HS Assay Kit and Qubit^®^ 2.0 fluorometer (Invitrogen, Thermo Fisher, Waltham, MA, USA). Droplet digital PCR (ddPCR) assays for four *TERT* promoter somatic mutations, i.e., the most prevalent C228T and C250T, and the rare C228A, CC242-243TT mutations have been previously developed and validated [32,42]. As rare A161C mutations have been described in bladder cancer cells [29], we further established the corresponding ddPCR assay. Probes and primers are described in Table S1.

For each ddPCR assay, 5′-FAM or 5′-HEX reporter dye and 3′ Iowa Black Fluorescent quencher were designed (Bio-rad, Hercules, CA, USA). A 22 µL reaction mix was prepared using 10 ng of DNA was used as a template, 11 µL of 2x ddPCR supermix-no dUTP (Bio-rad), 1.1 µL of 20x FAM and HEX probes for mutated and wild type alleles, 1.1 µL of RsaI restriction enzyme (10 U/µL) and 0.2 µL of 7-deaza-dGTP, Li-salt (2 µM). The droplets were generated using the AutoDG droplet generator (Bio-rad). The PCR amplification of the droplets was carried out separately for the C228T, C228A, CC242-243TT, and C250T assays using the following PCR conditions: 95 °C for 10 min, 40 cycles of 94 °C for 30 secs, ramp 2.5/sec, 54 °C for C228T assay (55 °C for the C228A and CC242-243TT, 62 °C for A161C and 64 °C for C250T assays) for 30 secs followed by 98 °C for 10 min and then kept at hold at 12 °C. The fluorescent intensity of each droplet was measured using the droplet reader QX200 (Bio-rad). Analysis of ddPCR data was performed using QuantaSoftTM Analysis Pro1.0.596 software (Bio-rad). The preparation of the droplets using the AutoDG, the subsequent PCR amplification, and the measurement of fluorescent intensity using the QX200 droplet reader were performed in three separate rooms specific for these respective purposes to ensure a contamination-free environment. The 2D amplitude plots from the QuantaSoftTM analysis pro software were analyzed by setting the threshold amplitudes for both the mutated and wild-type channels. The threshold for the minimum number of positive droplets for calling a mutation was determined from the Poisson distribution of droplets in wild-type and was set at 6 (C228T/CC242-243TT assays), 5 (C228A/C250T assays), and 3 (A161C assay). All laboratory analyses were conducted blindly to the case or control status. However, samples with a number of positive droplets under the thresholds were screened again with 20 ng input urinary DNA to evaluate whether the increased amount of DNA allowed discriminating the false-negative results to low-level mutated samples.

### 4.5. Statistical Analysis

We used Pearson’s chi-squared and Fisher’s exact tests to compare the dichotomous variables. We tested the distribution of the continuous variables by Shapiro–Wilk test. For normally distributed data, we used Student’s t-tests, and for the non-normally distributed data, we used the Mann–Whitney test to compare the differences between the continuous variables. We calculated the diagnostic tests’ sensitivity, specificity, accuracy, and confidence intervals using the Clopper–Pearson method [43]. To calculate the positive predictive value (PPV) and negative predictive values (NPV), we estimated a prevalence of 30% for BC in patients with hematuria, lower urinary tract symptoms, and other urological pathologies, which is based on previous work by Springer et al. [25]. Confidence intervals for PPV and NPV were calculated using the method of Mercaldo et al. [44]. All statistical analyses were performed using Stata statistical software version 16 (Stata Corporation, College Station, TX, USA).

## 5. Conclusions

In conclusion, our study validated the ability of ddPCR assays to detect the very low level of urinary *TERT* promoter mutations (uTERTpm) and demonstrated promising diagnostic accuracy of the uTERTpm marker for the detection of BC, in particular, primary BC in the Kerman province of Iran where the highest incidence of bladder cancer has been observed in Iran and where opium and tobacco consumption may largely contribute to the BC burden. Should the robustness of the uTERTpm marker be further validated in large case–control and screening studies, this may lead to a significant improvement in the way BC is detected and clinically managed. Particularly in LMICs where cystoscopy facilities may not be easily accessible and where increased incidence of BC has been recently observed in specific regions, a simple and inexpensive urine biomarker, such as uTERTpm, could provide an interesting non-invasive alternative for the triage of uTERTpm-positive individuals who would benefit from further diagnostic imaging. Finally, the recent discovery of the activation of the C228T TERT promoter allele in bladder cancer cells by the tripartite motif containing 28 (TRIM28) transcription factor which could be blocked by rapamycin analog opens new avenues for TERT-directed cancer therapies [45], possibly guiding in the future treatment decision making in patients with uTERTpm-positive tests and confirmed diagnosis of BC.

## Figures and Tables

**Table 1:** Distribution of demographics and selected exposures among participants in the case and control groups.

Characteristic	Case Group (Total *n* = 31) N (%)	Control Group (Total *n* = 50) N (%)	*p*-Value
**Age** (years) ^1^	64.9 ± 9.4	59.6 ± 13.4	0.058
**Sex**			
Male	26/31 (83.8%)	34/50 (74%)	*p* = 0.11
Female	5/31 (16.1%)	16/50 (26%)	
**Smoking**	26/31 (83%)	8/50 (16%)	*p* < 0.001
**Opium use**	24/31 (77%)	7/50 (14%)	*p* < 0.001
**Hematuria**			
Gross Microscopic	19/31 (61%) 9/31 (29%)	- -	-
**Tumor stage**			
MIBC ^2^	7/31 (22%)	-	-
NMIBC ^3^	24/31 (77%)	-	
**Tumor grade**			
Low-grade	16/31 (52%)	-	-
High-grade	15/31 (48%)	-	

^1^ Age is illustrated as median ± standard deviation; ^2^ MIBC: muscle-invasive bladder cancer; ^3^ NMIBC: non-muscle-invasive bladder cancer.

**Table 2:** Performance of uTERTpm in detecting primary and recurrent bladder cancers.

Characteristics	All BC Cases (*n* = 31)	Primary BC (*n* = 11)	Recurrent BC (*n* = 20)
True positive (n)	21	11	10
True negative (n)	44	44	44
False positive (n)	6	6	6
False negative (n)	10	0	10
Sensitivity (95% CI) (%)	67.7 (48.6–83.3)	100.0 (71.5–100.0)	50.0 (27.2–72.8)
Specificity (95% CI) (%)	88.0 (75.6–95.4)	88.0 (75.6–95.4)	88.0 (75.6–95.4)
Positive likelihood ratio (95% CI)	5.6 (2.5–12.4)	8.3 (3.9–17.6)	4.1 (1.7–9.9)
Negative likelihood ratio (95% CI)	0.3 (0.2–0.6)	0.0	0.5 (0.3–0.8)
Positive predictive value (95% CI) (%) *	70.7 (52.3–84.1)	78.1 (62.7–88.3)	64.1 (42.8–80.9)
Negative predictive value (95% CI) (%)*	86.4 (79.0–91.4)	100.0	80.4 (72.3–86.5)
Accuracy (95% CI) (%) *	81.9 (71.8–89.6)	91.6 (81.6–97.1)	76.6 (64.9–85.9)

* Positive and negative predictive values were calculated for patients at high risk of developing bladder cancer, estimated at 30% for patients with hematuria or patients with lower urinary tract symptoms or others, according to Springer et al. (2018) [25].

**Table 3:** Performance of uTERTpm and cytology in detecting primary and recurrent bladder cancers across different subgroups of patients.

Subgroups	uTERTpm		Urine Cytology		Combined uTERTpm/Urine Cytology	
	Sensitivity % (95% CI)	Specificity % (95% CI)	Sensitivity % (95% CI)	Specificity% (95% CI)	Sensitivity % (95% CI)	Specificity% (95% CI)
**All patients**	67.7 (48.6–83.3)	88.0 (75.6–95.4)	67.7 (48.6–83.3)	62.0 (47.1–75.3)	83.8 (66.2–94.5)	52.0 (37.4–66.3)
**Tumor stage**						
NMIBC (*n* = 24)	66.7 (44.7–84.4)	-	58.3 (36.7–77.9)	-	79.2 (57.9–92.9)	-
MIBC (*n* = 7)	71.4 (29.0–96.3)	-	100.0 (59.0–100.0)	-	100.0 (59.0–100.0)	-
**Grade**						
Low-grade (*n* = 16)	62.5 (35.4–84.8)	-	56.2 (29.9–80.3)	-	75.0 (47.6–92.7)	-
High-grade (*n* = 15)	73.3 (44.9–92.2)	-	80.0 (51.9–95.7)	-	93.3 (68.1–99.9)	-
**Gross Hematuria**						
Yes (*n* = 19)	73.6 (48.8–90.8)	-	57.8 (33.5–79.6)	-	84.2 (60.4–96.6)	-
No (*n* = 12)	58.3 (27.6–84.8)	-	83.3 (51.5–97.9)	-	83.3 (51.5–97.9)	-
**Smoking**						
Yes (*n* = 25)	69.2 (48.2–85.6)	77.7 (39.9–97.1)	76.9 (56.3–91.0)	66.6 (29.9–92.5)	88.4 (69.8–97.5)	44.4 (13.7–78.8)
No (*n* = 6)	60.0 (14.6–94.7)	90.2 (76.8–97.2)	20.0 (0.5–71.6)	60.9 (44.5–75.8)	60.0 (14.6–94.7)	53.0 (37.4–69.3)
**Opium use**						
Yes (*n* = 23)	75.0 (53.2–90.2)	85.7 (42.1–99.6)	70.8 (48.9–87.3)	57.1 (18.4–90.1)	87.5 (67.6–97.3)	42.8 (9.9–81.5)
No (*n* = 8)	42.8 (9.9–81.5)	88.3 (74.9–96.1)	57.1 (18.4–90.1)	62.7 (46.7–77.0)	71.4 (29.0–96.3)	53.4 (37.6–68.8)

## Data Availability

Detailed uTERTpm screening and clinical data are available upon request to corresponding authors.

## Supplementary Materials

The following supporting information can be downloaded at: https://www.mdpi.com/article/10.3390/ijms232214319/s1.
