# Supplementary material for: TERT Promoter Mutations as Simple and Non-Invasive Urinary Biomarkers for the Detection of Urothelial Bladder Cancer in a High-Risk Region
Source: Int J Mol Sci. 2022 Nov 18;23(22):14319. doi: 10.3390/ijms232214319 (PMC9696845; doi:10.3390/ijms232214319)
Supplement: Supplementary file 1 [file ijms-23-14319-s001.zip › ijms-1886337-supplementary.pdf]

Table S1. ddPCR probes and primers for detecting TERT promoter mutations

Mutation type	Primer/probe	Sequence (5’ to 3’)	Fluorescent Dye and quencher	PCR Product Size (bp)
TERT C228T	fw_primer	CCCTCCCGGGTCC	-	64
	rev_primer	CCGCGGAAAGGAAGG	-	
	wt_probe	CGGAgGGGGCTGG	HEX_IowaBlack	
	mut_probe	CCCGGAaGGGGCTG	FAM_IowaBlack	
TERT C250T	fw_primer	TCCAGCTCCGCCTCCTCC	-	109
	rev_primer	GGGCCGCGGAAAGGAAGG	-	
	wt_probe	TCCCGACCCCTcCCGGGTCC	HEX_IowaBlack	
	mut_probe	TCCCGACCCCTtCCGGGTCC	FAM_IowaBlack	
TERT C228A	fw_primer	CGCGGAAAGGAAGGG	-	64
	rev_primer	CCCCTCCCGGGTC	-	
	wt_probe	CGGAgGGGGCTGG	HEX_IowaBlack	
	mut_probe	CCCGGAtGGGGCTG	FAM_IowaBlack	
TERT CC242-243TT	fw_primer	GAGGGCCCGGAGG	-	88
	rev_primer	CTTCACCTTCCAGCTCC	-	
	wt_probe	CTGGGCCGGggAC	HEX_IowaBlack	
	mut_probe	CCGGaaACCCGGGA	FAM_IowaBlack	
TERT A161C	fw_primer	CGGACCCCGCCCCGT	-	154
	rev_primer	CCAGGGCTTCCCACGTGC	-	
	wt_probe	CAGCGCTGCCGGAAACTCG	HEX_IowaBlack	
	mut_probe	CAGCGCTGCCTGAAACTCGC	FAM_IowaBlack	

Fw: Forward; rev: Reverse; wt: Wild-type; mut: Mutated.

ddPCR probes containing either a 5′-FAM or 5′-HEX reporter dye and 3′ Iowa Black® Fluorescent quencher were HPLC purified.

Table S2. Distribution of demographics and selected exposures among patients with primary and recurrent bladder cancer

**Characteristic**	**Primary BC (total n=11) N (%)**	**Recurrent BC (total n= 20) N (%)**	**P-value**
**Age** (years) [1]	62.2 ± 7.22	66.05 ± 10.5	0.301
**Sex**			1.000
Male	9 (81%)	17 (85%)	
Female	2 (18%)	3 (15%)	
**Active smoking**	11 (100%)	15 (75%)	0.070
**Opium use**	10 (91%)	14 (70%)	0.183
**Hematuria**			0.173
Gross	9 (81%)	10 (50%)	
Microscopic	2 (18%)	7 (35%)	
**Tumor stage**			0.676
MIBC[2]	3 (27%)	4 (20%)	
NMIBC[3]	8 (73%)	16 (80%)	
**Tumor grade**			0.809
Low grade	6 (54%)	10 (50%)	
High grade	5 (45%)	10 (50%)	
**Urine cytology**			1.000
Positive	8 (73%)	13 (65%)	
Negative	3 (27%)	7 (35%)	

1: Age is illustrated as median ± standard deviation

2. MIBC: Muscle-invasive bladder cancer

3. NMIBC: Non Muscle-invasive bladder cancer

Table S3. Distribution of the subtypes of TERT promoter mutations detected by ddPCR among participants in the case and control groups

**Mutation type**	**Case group (total n=31) N (%)**	**Control group (total n= 50) N (%)**	**P-value**
TERT C228T	18 (58%)	4 (8%)	<0.001
TERT C250T	4 (13%)	1 (2%)	0.068
TERT C228A	0	0	-
TERT CC242-243TT	0	0	-
TERT A161C	0	1 (2%)	0.617
Any of the above	20 (64.5%)	6 (12%)	<0.001

Figure S1. Sanger sequencing Chromatogram of the *TERT* promoter sequence of the KER-0023 urinary DNA sample identifying the C158A mutation.

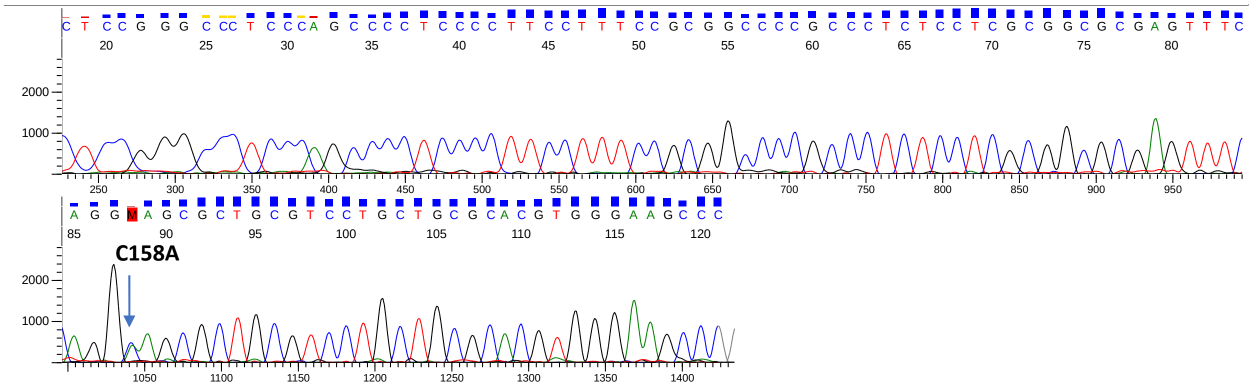

The Mutant Allelic Fraction (MAF) of the C158A in the urinary DNA of this BC case is estimated to be at 50%.
